# Influences of the Culturing Media in the Virulence and Cell Wall of *Sporothrix schenckii*, *Sporothrix brasiliensis*, and *Sporothrix globosa*

**DOI:** 10.3390/jof6040323

**Published:** 2020-11-28

**Authors:** Nancy E. Lozoya-Pérez, Diana M. Clavijo-Giraldo, Iván Martínez-Duncker, Laura C. García-Carnero, Luz A. López-Ramírez, Gustavo A. Niño-Vega, Héctor M. Mora-Montes

**Affiliations:** 1 Departamento de Biología, División de Ciencias Naturales y Exactas, Campus Guanajuato, Universidad de Guanajuato, Noria Alta s/n, col. Noria Alta, C.P., Guanajuato Gto. 36050, Mexico; nelppat@hotmail.com (N.E.L.-P.); diamar438@hotmail.com (D.M.C.-G.); laura_cgc@hotmail.com (L.C.G.-C.); adrianalr@ugto.mx (L.A.L.-R.); gustavo.nino@ugto.mx (G.A.N.-V.); 2 Laboratorio de Glicobiología Humana y Diagnóstico Molecular, Centro de Investigación en Dinámica Celular, Instituto de Investigación en Ciencias Básicas y Aplicadas, Universidad Autónoma del Estado de Morelos, Cuernavaca Mor. 62209, Mexico; duncker@uaem.mx

**Keywords:** sporotrichosis, lactate dehydrogenase, invertebrate infection model, hemocytes, phenoloxidase, glucan, chitin, glycoprotein

## Abstract

*Sporothrix schenckii*, *Sporothrix brasiliensis*, and *Sporothrix globosa* are etiological agents of sporotrichosis, a human subcutaneous mycosis. Although the protocols to evaluate *Sporothrix* virulence in animal models are well described, the cell preparation before inoculation is not standardized, and several culturing media are used to grow yeast-like cells. Here, we found that carbon or nitrogen limitation during fungal cell preparation negatively impacted the ability of *S. schenckii* and *S. brasiliensis* to kill *Galleria mellonella* larvae, but not *S. globosa*. The fungal growth conditions associated with the short median survival of animals were accompanied by increased hemocyte countings, phenoloxidase activity, and cytotoxicity. The fungal growth under carbon or nitrogen limitation also affected the cell wall composition of both *S. schenckii* and *S. brasiliensis* and showed increased exposure of β-1,3-glucan at the cell surface, while those growing conditions had a minimal impact on the *S.* *globosa* wall, which had higher levels of this polysaccharide exposed on the wall regardless of the culture condition. This polysaccharide exposure was linked to the increased ability of insect hemocytes to uptake fungal cells, suggesting that this is one of the mechanisms behind the lower virulence of *S.* *globosa* or cells from the other species grown in carbon or nitrogen limitation.

## 1. Introduction

Superficial, subcutaneous, and deep-seated mycoses are a frequent human health problem worldwide, and the morbidity and mortality rates associated with systemic infections are continuously increasing, despite the availability of antifungal drugs and different treatment schemes [1,2]. Sporotrichosis is a subcutaneous infection distributed worldwide that affects humans and other mammals, particularly domestic species such as cats and less frequently dogs [3,4,5]. Even though many cases can be self-limited and treated with conventional antifungal therapies, some can become systemic infections with high mortality rates; in particular, if the host is immunocompromised [3,5]. This disease is caused by members of the *Sporothrix* genus, a taxonomic group that includes environmental species as well as pathogens of insects and mammals [3,6]. The newest taxonomical proposal of the *Sporothrix* species includes the grouping in clades, and the pathogenic clade harbors the species most frequently isolated from human and veterinary sporotrichosis cases, named *Sporothrix schenckii*, *Sporothrix brasiliensis*, and *Sporothrix globosa* [6]. *S. schenckii* is the first species recognized as an etiological agent of this disease [3,7] and is distributed worldwide [8]. *S. brasiliensis* has been thus far only reported in Brazil and Argentina [3,9,10], is frequently associated with feline sporotrichosis and is associated with epidemic outbreaks in both cats and humans, in some Brazilian areas [9,11]. Due to the high incidence of feline disease and close contact with humans, this infection is now considered zoonotic [3]. *S. globosa* has also been found as an etiological agent of human sporotrichosis, but it is a species with low molecular diversity, whose main prevalence is in Asia and to a lesser extent in America [8,12,13]. Both genomic and phenotypical analyses of these species have highlighted that they have species-specific traits that might contribute to explain the pathogenicity of these species, the clinical presentation of the infections, geographical distributions, and responses to treatments [12,14,15,16,17,18]. *S. brasiliensis* has been reported as the most virulent species of the pathogenic clade, associated with severe clinical forms of the mycosis, with zoonotic outbreaks, high mortality rates, and tissue burden in animal models, while *S. schenckii* often causes benign chronic subcutaneous infections, with a moderate virulence in animal models, and *S. globosa* is reported as a low-virulence species responsible for sapronosis [3,19]. The antifungal susceptibility has been reported to be a species-specific trait. *S. brasiliensis* has the best response to antifungals, followed by *S. schenckii*, whereas *S. globosa* is less susceptible to most of the antifungals tested, such as amphotericin B, ketoconazole, posaconazole, and itraconazole [20]. Moreover, animal-isolated strains have lesser azoles susceptibility than those recovered from human samples [21].

The virulence in laboratory animals is one of the aspects most frequently addressed when studying a fungal pathogen. This strategy, combined with chemical treatments or pathogen genetic manipulation, helps to unveil virulence factors and determinants and to assess their contribution to the pathogen aggressivity and resilience. In the case of the *Sporothrix* species, mice have been chosen as the model organism to assess virulence, and the most widely used experimental infection involves the subcutaneous administrations of fungal cells [22,23,24,25,26,27,28]. Intraperitoneal and intravenous models have also been standardized to assess the *Sporothrix* virulence [17,19,26,29,30,31]. These strategies have allowed establishing the virulence ranking among the most relevant members of the pathogenic clade of *Sporothrix* [17,19,25,29]. Additionally, differences in the virulence of isolates of the same species have been reported [17,19,31].

Invertebrates have risen as an alternative to evaluate fungal virulence, and a model of experimental sporotrichosis in larvae of *Galleria mellonella* has been reported [32,33,34]. The use of this organism has the advantage that it requires simple housing and breeding facilities, and due to the substantial numbers of animals bred by generation, it is possible to include large numbers of individuals in the experimental population, providing statistical strength. [32,35,36]. *G. mellonella* larvae stand out from the other invertebrates because they can grow at 37 °C, allowing the study of *Sporothrix* thermodimorphic traits [35,37]; possess immunological cells, named hemocytes, that are found in the hemolymphand can perform fungal phagocytosis like mammalian macrophages [35,38,39].

Although the animal model to assess the *Sporothrix* spp. virulence is well standardized in both mice and *G. mellonella*, there is no consensus on the fungal morphology and inoculum preparation to challenge the animal model. The most frequently reported *Sporothrix* morphology to prepare cells for animal inoculation is yeast-like cells grown in brain–heart infusion (BHI) [24,25,30,31,40], although yeast-like cell preparations have also been reported in yeast–peptone–dextrose (YPD) medium [32,33,34], Sabouraud broth [29], and potato-dextrose broth [17]. Conidia have also been used to inoculate *Sporothrix* cells in the animal models, and these are reported to be harvested from cultures growing on mycosel, Sabouraud, and potato-dextrose-agar plates [19,23,26,28]. There is evidence in *Candida albicans* indicating that the culture media to prepare cells has a strong influence on the phenotypical trait that will contribute to damage and kill the animal model [41,42,43,44,45,46].

Here, we explored the virulence of *S. schenckii*, *S. brasiliensis,* and *S. globosa* yeast-like cells grown in different culture media and found that these have a significant impact on the ability of *S. schenckii* and *S. brasiliensis* to kill larvae, but not in the case of *S. globosa*. Moreover, we assessed the effect of the culture media on the fungal cell wall composition and the ability of insect hemocytes to uptake fungal cells.

## 2. Materials and Methods

### 2.1. Strains and Culture Conditions

The strains used in this study are *S. schenckii* 1099-18 (ATCC MYA 4821) and *S. brasiliensis* 5110 (ATCC MYA 4823), clinical isolates whose genome has been previously sequenced [15,27], along with *S. globosa* FMR 9624, a clinical isolate from human lymphocutaneous sporotrichosis previously characterized [47]. Cells were maintained and propagated at 28 °C in YPD medium (2% (*w*/*v*) gelatin peptone, 1% (*w*/*v*) yeast extract, and 3% (*w*/*v*) glucose). After cell confluency, typically 7 days, conidia were scrapped out from the agar surface, with the cell concentration quantified and used to inoculate 20 mL fresh YPD broth, pH 7.8, at a concentration of 1 × 10^6^ conidia/mL. The cultures were incubated in orbital shakers at 37 °C and 120 rpm for 18 h. Then, an aliquot of 10 mL was removed from these cultures, cells were sedimented by centrifuging at 2700× *g* and 4 °C for 10 min, washed twice with PBS, and inoculated in 50 mL of the culture media to induce dimorphism [14]. These culture media were YPD pH 7.8, BHI (Oxoid-Thermo Scientific, Hampshire, United Kingdom), YP pH 7.8 (2% (*w*/*v*) gelatin peptone and 1% (*w*/*v*) yeast extract), and YNB pH 7.5 (0.67% (*w/v*) yeast nitrogen base with amino acids and 2% (*w/v*) glucose). The cultures were incubated at 37 °C and 120 rpm for four days to stimulate the transition to yeast-like cells [14,48]. For the cases where dimorphism did not occur in nearly 100% of cells, yeast-like cells were separated from mycelia by filtrating in a Buchner filter, as described [49], and used to inoculate *G. mellonella* larvae

### 2.2. Galleria mellonella Survival Assays

The *G. mellonella* larvae were from an in-house colony previously established [32] and were fed ad libitum on corn bran and honey (1 kg corn bran, 150 g rice meal, 250 mL bee honey, and 70 mL glycerin) [50]. The inclusion criteria for the use of larvae in this experimental setting were the absence of evident body injuries or melanization and a size of 1.2–1.5 cm [32,50]. The last left pro-leg was cleansed with 70% (*v*/*v*) ethanol 1 × 10^5^ yeast-like cells, contained in 10 μL of PBS, and injected with a Hamilton syringe equipped with a 26-gauge needle [32]. The infected larvae were grouped by experimental condition, kept at 37 °C in Petri dishes, and monitored daily for survival. To avoid animal dehydration, chopped apple was included in the animal housing [32,50]. To delay the transition to the pupa, the silk on the animal surface was removed. Lack of irritability and extensive body melanization were both taken as animal death indicators. As a control, animals were injected only with PBS, to assess the mortality by animal manipulation and mechanical injuries. Each group, including the control, was composed of 30 larvae. To calculate the colony-forming units (CFUs), alive and dead animals were decapitated with a sterile scalpel, and the hemolymph was serially diluted and incubated on YPD plates, pH 4.5, at 28 °C for 72 h.

### 2.3. Ethics Statement

The use of animals in this study was approved by the Ethics Committee of Universidad de Guanajuato (permission CIBIUG-P12-2018).

### 2.4. Analysis of Hemocyte Levels, Phenoloxidase, and Lactate Dehydrogenase Activities

Groups of 10 animals were inoculated as described in Section 2.2 and incubated at 37 °C for 24 h before decapitation. On average, 30 µL of hemolymph were recovered from each larva and mixed with 150 µL of anticoagulant solution (93 mM NaCl, 100 mM glucose, 30 mM trisodium citrate, 26 mM citric acid, 10 mM Na_2_EDTA, and 0.1 mM phenylthiourea, pH 4.6) [51]. To reduce cell clumping, samples were stored on ice and processed the same day. Hemocytes were quantified in a hemocytometer, as described elsewhere [52]. Phenoloxidase activity was determined as previously reported [53]. The hemolymph was centrifuged at 20,000× *g* for 10 min, the supernatant was saved, and protein concentration was quantified with the Pierce BCA Protein Assay (Thermo Fisher Scientific, Waltham, MA, USA). The enzyme reactions were performed in 200 µL and contained 100 µg protein and 20 mM 3,4-dihydroxyDL-phenylalanine (Sigma-Aldrich St. Louis, MO, USA). They were placed in 96-well microplates, and the initial absorbance at 490 nm was read in a MultiskanTM FC microplate photometer (Thermo Fisher Scientific, Waltham, MA, USA). The reactions were incubated for 30 min at 37 °C, and the absorbance at the same wavelength was measured again. Enzyme activity was defined as the change in the absorbance at 490 nm per minute and per µg protein [51]. The released lactate dehydrogenase (LDH) activity in the cell-free hemolymph was analyzed with the Pierce LDH Cytotoxicity Assay (Thermo Fisher Scientific, Waltham, MA, USA), and the absorbances at 490 nm and 680 nm were obtained using a MultiskanTM FC microplate photometer (Thermo Fisher Scientific, Waltham, MA, USA) [54]. The 100% cytotoxicity was the LDH activity of fresh cell homogenates, and samples from non-infected larvae were used as controls.

### 2.5. Cell Wall Analysis

To determine the wall carbohydrate composition, yeast-like cells were disrupted in a Braun homogenizer as described previously [14,49]. The homogenates were centrifuged at 8000× *g* for 15 min to remove the soluble fraction, and the pellet was washed six times with deionized water. Intracellular components were removed from the cell wall preparations by serial incubations with hot SDS, β-mercaptoethanol, and NaCl before hydrolysis with 2 M trifluoroacetic acid (Sigma-Aldrich, St. Louis, MO, USA), as reported elsewhere [55]. The acid-hydrolyzed samples were analyzed by high-performance anion-exchange chromatography with pulsed amperometric detection (HPAEC-PAD) in a Dionex system (Thermo Fisher Scientific, Waltham, MA, USA) using a CarboPac PA-1 column and the elution conditions described earlier [56]. The cell wall protein content was determined in alkali-hydrolyzed walls with 1 N NaOH, as described [55].

To assess the ability to bind Alcian blue, yeast-like cells were pelletized and washed twice with deionized water, and the cell suspension was adjusted at an OD_600nm_ of 0.2 in deionized water. Aliquots of 1 mL were centrifuged at 9485× *g* for 2 min, the supernatant was discarded, and the cells were suspended in 1 mL of Alcian blue (Sigma-Aldrich, St. Louis, MO, USA; 30 μg/mL, in 0.02 M HCl). The cell suspension was incubated at room temperature for 10 min, centrifuged at 9485 × *g* for 2 min, the absorbance at 620 nm was measured, and the bound dye was quantified as described previously [57].

The cell wall *N*-linked and *O*-linked glycan content was determined as described elsewhere [58]. To remove *O*-linked glycans, yeast-like cells were suspended in 1 N NaOH and gently shaking for 18 h at room temperature; while *N*-linked glycans were trimmed by incubating cells with 25 U endoglycosidase H (New England Biolabs; Ipswich, MA, USA) at 37 °C for 20 h. Glycan trimming was confirmed by HPAEC-PAD, following the separation conditions previously reported [59], and quantification was performed by the phenol-sulfuric acid method [60].

For the β-1,3-glucan fluorescent labeling, cells were incubated with 5 μg/mL IgG Fc-Dectin-1 chimera [61] for 40 min at room temperature and then with 1 μg/mL donkey anti-Fc IgG-fluorescein isothiocyanate (Sigma-Aldrich, St. Louis, MO, USA) for 40 min at room temperature [62], while chitin labeling was performed by incubating cells with 1 mg/mL wheat germ agglutinin-fluorescein isothiocyanate (Sigma-Aldrich St. Louis, MO, USA) for 60 min at room temperature [63]. Samples were inspected under fluorescence microscopy using a Zeiss Axioscope-40 microscope and an Axiocam MRc camera. The fluorescent associated with 300 cells per condition was estimated as reported [64]. The heat-killed (HK) cells were prepared by incubating at 60 °C for 2 h [14]. The cellular death was confirmed by incubating aliquots of the preparations in YPD plates at 28 °C for 5 days.

### 2.6. Analysis of Uptake by Hemocytes and Fungal-Secreted Protease and Lipase/Esterase Activities

For analysis of fungal uptake, yeast-like cells were washed with PBS and labeled with 1 mg/mL acridine orange (Sigma-Aldrich St. Louis, MO, USA) as described previously [65]. Then, cells were washed twice with PBS, cell concentration adjusted at 3 × 10^7^ yeast cells/mL and deposited in six-well plates, and the interactions were performed with a hemocyte–yeast ratio of 1:6 in 800 µL of DMEM (Sigma-Aldrich, St. Louis, MO, USA). Fresh hemocytes were collected from healthy larvae and mixed with the anticoagulant solution as described in Section 2.4. Plates were incubated for 2 h at 37 °C and 5% (*v/v*) CO_2_, and hemocytes washed twice with cold PBS and suspended in 1.25 mg/mL trypan blue as an external fluorescence quencher [66]. Samples were analyzed by flow cytometry in a FACSCanto II equipped with a FACSDiva acquisition system (Becton Dickinson, Franklin Lakes, NJ, USA), collecting 25,000 events gated for hemocyte cells. Fluorescent signals were obtained using the FL1 (green) and FL2 (red) channels previously compensated with non-stained hemocytes [65,66]. When required, hemocytes were preincubated with 200 µg/mL laminarin (Sigma-Aldrich, St. Louis, MO, USA) for 1 h at 37% and 5% (*v/v*) CO_2_ before adding to the interaction with yeast-like cells. Although laminarin was negative to bacterial lipopolysaccharide presence (determined with the *Limulus* amebocyte lysate from Sigma-Aldrich, St. Louis, MO, USA), the preincubations with this compound were performed in the presence of 5 mg/mL polymyxin B (Sigma-Aldrich, St. Louis, MO, USA) [67]. To allow the appropriate comparison, the system with no preincubation with laminarin was also performed in presence of this drug.

Protease activity was measured in reactions containing 10 mM Tris-HCl, pH 7.5, 1 × 10^5^ yeast-like cells, and 1.0 mg bovine serum albumin (Thermo Fisher Scientific Waltham, MA, USA). Reactions were incubated at 37 °C for 60 min; then, cells were pelleted by centrifuging at 9485 × *g* for 2 min, the supernatant was saved, enzyme activity was inactivated by boiling for 10 min and filtrated in an Amicon^®^ ultra-4 centrifugal filter unit—3K NMWL (Merck KGaA, Darmstadt, Germany)—at 4000 × *g* for 15 min at room temperature, and the eluted samples were saved and used to determine the amino acid concentration by Bradford’s method. For data normalization, 1 × 10^5^ yeast-like cells were suspended in 10 mM Tris-HCl, pH 7.5, and incubated for 60 min, and the cell-free fraction was used to quantify total protein content. A similar reaction was processed by ultrafiltration, and the amino acid content in the eluted sample was subtracted from the reading of the assays where albumin was included. As negative controls, mock reactions with no cell suspension were included. As a positive control, 10 U proteinase K (Thermo Fisher Scientific Waltham, MA, USA) was suspended in 10 mM Tris-HCl, pH 7.5, and 0.2% (*v/v*) SDS, mixed with 1.0 mg bovine serum albumin, and processed as above described.

For lipase/esterase activity, 1 × 10^5^ yeast-like cells were suspended in 10 mM Tris-HCl, pH 7.5, 0.04 mM 4-methylumbelliferyl-oleate (Sigma-Aldrich, St. Louis, MO, USA) was added, and reactions incubated at 37 °C for 60 min. Then, cells were pelleted by centrifuging at 9485× *g* for 2 min, the supernatant was saved, enzyme activity was inactivated by boiling for 10 min, and the released 4-methylumbelliferone was measured in a Perkin-Elmer LS-5B luminescence spectrofluorometer, with excitation and emission set at 350 nm and 440 nm, respectively [68]. Mock reactions, with no cells added, were included as negative controls. As a positive control, 10 U pig pancreatic lipase (Sigma-Aldrich, St. Louis, MO, USA) and 0.04 mM 4-methylumbelliferyl-oleate were mixed, incubated at 37 °C for 60 min, and processed as described above.

### 2.7. Statistical Analysis

Statistical analysis was performed using the GraphPad Prism 6 software. Survival experiments were performed with a total of 30 larvae per group, and data were plotted in Kaplan–Meier survival curves and analyzed using the log-rank test. The Mann–Whitney U test was used to analyze other results, which are reported as the media ± standard deviation from three independent experiments performed by duplicate. In all cases, the statistical significance was set at *p* < 0.05.

## 3. Results

### 3.1. The Culture Medium Affects the Sporothrix *spp.* Ability to Kill Galleria mellonella Larvae

Conidia were grown in either YPD or BHI broth to undergo dimorphism as described in the Materials and Methods section. Under these conditions, more than 95% of cells were yeast-like cells for *S. schenckii*, *S. brasiliensis*, and *S. globosa*. The dimorphism was also stimulated in YP and YNB broth, as the former lacks a carbon source additional to that provided by the peptone and the yeast extract, and the latter contains a lower nitrogen amount compared to the peptone-based media. In YP, cells showed a significantly different ability to undergo dimorphism (*p* < 0.05), with 54.3 ± 11%, 82.2 ± 9%, and 94.2 ± 3% of yeast-like cells in the cultures of *S. schenckii*, *S. brasiliensis*, and *S. globosa*, respectively. In YNB, 35.4 ± 8%, 60.3 ± 7%, and 90.2 ± 5% of cells were converted into yeast-like cells for *S. schenckii*, *S. brasiliensis*, and *S. globosa*, respectively, and these differences were statistically significant (*p* < 0.05).

**Figure 1:**
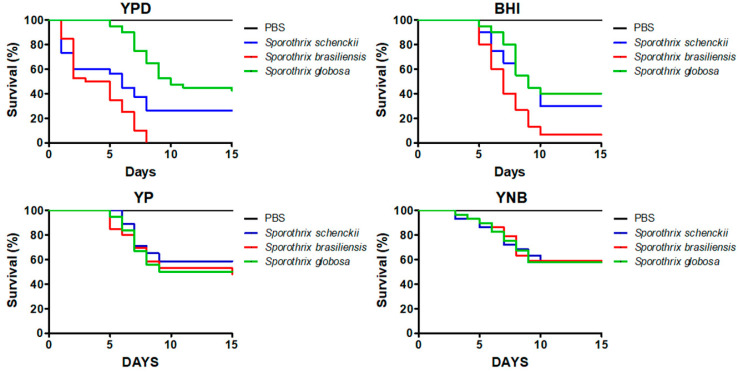
Mortality curves of *Galleria mellonella* larvae infected with *Sporothrix schenckii*, *Sporothrix brasiliensis,* or *Sporothrix globosa*. The yeast-like cells were grown in yeast–peptone–dextrose (YPD), brain–heart infusion (BHI), YP, or YNB media and were used to inoculate 1 × 10^5^ yeast-like cells per animal. Each group contained 30 larvae (10 larvae for each experiment). Larvae were monitored daily to assess survival, which was defined by the presence of irritability and the absence of extensive body melanization. PBS, control group injected only with PBS.

The survival curves of animals infected either with *S. schenckii* or *S. brasiliensis* yeast-like cells grown in YPD were similar to those previously reported [32], with a median survival of 6.0 ± 1.1 and 4.0 ± 1 days for *S. schenckii* and *S. brasiliensis,* respectively (Figure 1). Even though the *S. globosa* virulence has not been previously demonstrated in *G. mellonella* larvae, following the same protocol used for the other two species under analysis, we found that the median survival of animals inoculated with this strain was 10.0 ± 1.2 days (Figure 1), with the three species showing a significantly different ability to kill *G. mellonella* (*p* < 0.05). When a similar experiment was performed with cells grown in BHI, we found that the median survival for animals infected with *S. schenckii*, *S. brasiliensis,* or *S. globosa* yeast-like cells were 9.0 ± 1.2, 7.0 ± 1.5, and 9.0 ± 1.3 days, respectively (Figure 1). The survival curves associated with *S. schenckii* and *S. globosa* were similar (*p* = 0.45), but significantly different from those generated when *S. brasiliensis* was inoculated (*p* < 0.05). The survival curves of animals infected with yeast-like cells grown in YP were similar (*p* = 0.78), with a median survival of 7.0 ± 1.1, 10.0 ± 1.4, and 9.0 ± 1.3 days for animals inoculated with *S. schenckii*, *S. brasiliensis*, or *S. globosa*, respectively (Figure 1). Similarly, cells grown in YNB showed similar abilities to kill the animal population (*p* = 0.99), with a median survival of 11 days for the three species under analysis (Figure 1).

**Figure 2:**
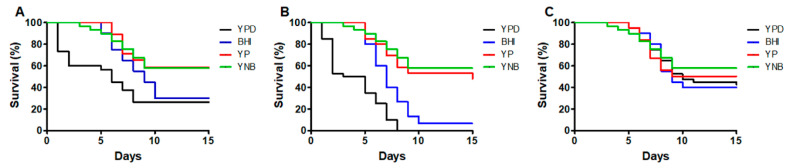
Comparison of the *Sporothrix* spp. ability to kill *Galleria mellonella* larvae. The data are the same as shown in Figure 1, but in this case, the mortality curves are grouped in one single chart for *Sporothrix schenckii* (**A**), *Sporothrix brasiliensis* (**B**), and *Sporothrix globosa* (**C**).

When the ability to kill the animal population of the same species grown in different conditions was analyzed, we found that the survival curves of animals inoculated with *S. schenckii* yeast-like cells grown in YPD or BHI were similar to each other (*p* = 0.14; Figure 2A), killing faster the animal population than cells grown in YP or YNB (*p* < 0.05; Figure 2A). In the case of animals infected with *S. brasiliensis*, only the curves generated with cells grown in YP or YNB were similar, with the others being significantly different among them and with shorter median survival (*p* < 0.05; Figure 2B). Finally, the survival curves generated with *S. globosa* cells grown in the four culturing media analyzed did not show any difference (*p* = 0.72; Figure 2C). Collectively, these data suggest that the *Sporothrix* ability to kill *G. mellonella* larvae is influenced by the culturing media, in a species-specific manner.

Next, we quantified the colony-forming units (CFU) recovered from the hemolymph of infected animals and found that this parameter did not change significantly in the animals infected with the different fungal species grown in the culture media under study (Table 1), suggesting that the fungal cells had similar abilities to colonize and adapt to the host milieu. The hemocyte quantification and phenoloxidase activity in the hemolymph have been associated with the host immunological response against a fungal pathogen [50,51,54], and the LDH activity in the cell-free hemolymph has been used as a cytotoxicity parameter that correlates with the fungal virulence [50,54]. In animals infected with *S. schenckii* yeast-like cells, the cytotoxicity varied depending on the culture media used to stimulate fungal dimorphism, with YPD-grown cells causing the highest cytotoxicity in animals, followed by fungal cells grown in BHI (Table 1). Cells grown in either YP or YNB caused the lowest and similar cytotoxicity (Table 1). A similar trend was observed in animals inoculated with *S. brasiliensis* cells grown in different culturing media, but for the case of larvae infected with *S. globosa*, the yeast-like cells induced similar cytotoxicity levels regardless of the culture medium used for cell growth (Table 1). The cytotoxicity found in animals infected with YPD- or BHI-grown cells was significantly lower in larvae infected with *S. globosa* than that observed in animal groups infected with either *S. schenckii* or *S. brasiliensis* (*p* < 0.05). For hemocyte levels and phenoloxidase activity in the hemolymph from *S. schenckii*-infected larvae, the highest levels of both parameters were observed in preparations from animals infected with YPD-grown cells, followed by the group infected with BHI-grown cells (Table 1). The animal groups infected with YP- or YNB-grown cells showed the lowest and similar values for the two immunological parameters (Table 1). Again, a similar trend was observed for the hemocyte levels and the phenoloxidase activity found in animals infected with *S. brasiliensis* cells, with the highest values associated with cells grown in YPD followed by BHI, and the lowest values found in animals infected with YP- or YNB-grown cells (Table 1). For the case of the animal groups infected with *S. globosa*, the culturing media used for cell preparation did not influence the hemocyte counting or the phenoloxidase activity found in the hemolymph (Table 1). The levels found in the animals infected with *S. globosa* YPD- or BHI-grown cells were significantly lower than those observed in animals infected with either *S. schenckii* or *S. brasiliensis* grown in the same culture medium (*p* < 0.05). Therefore, the animals infected with *S. schenckii* or *S. brasiliensis*, but not those with *S. globosa*, showed variations in the cytotoxicity, hemocyte counting, and phenoloxidase activity, depending on the culture medium used to prepare the fungal inoculum.

### 3.2. The Culture Medium Affects Sporothrix *spp.* Cell Wall Composition

**Figure 3:**
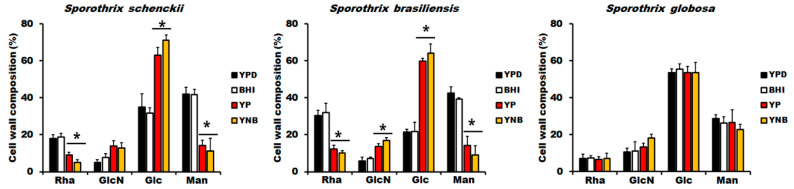
Cell wall composition of *Sporothrix schenckii*, *Sporothrix brasiliensis*, and *Sporothrix globosa* grown in different culture media. Yeast-like cells grown in indicated media were collected, and cell walls were isolated, cleaned, acid-hydrolyzed, and analyzed by high-performance anion-exchange chromatography with pulsed amperometric detection, as described in the Materials and Methods section. Cell walls preparations were from *Sporothrix schenckii*, *Sporothrix brasiliensis*, and *Sporothrix globosa*. Results are the media ± standard deviation from three independent experiments performed by duplicate. * *p* < 0.05 when compared to the sugar content of YPD- or BHI-grown cells.

As mentioned, in *C. albicans,* it has been demonstrated that the culturing conditions affect both the virulence and cell wall composition [43,45]. Therefore, we hypothesized that the growth in the media analyzed here could have an impact on the *Sporothrix* spp. cell wall composition. The cell walls from the yeast-like cells grown in the four culture media were collected and acid-hydrolyzed, and the monosaccharide composition was analyzed, as previously reported [14,33,34,48]. Glucose and glucosamine are regarded as the monomers of glucans and chitin, respectively, while rhamnose and mannose compose the glycans of cell wall glycoproteins [33,48]. In the case of yeast-like cells from *S. schenckii* and *S. brasiliensis* grown in YPD, the sugar composition was similar to that previously reported [14,48], with low rhamnose and higher glucose content in the *S. schenckii* cell wall than in *S. brasiliensis* (17.9 ± 2.1% vs. 30.4 ± 2.8% and 35.0 ± 7.1% vs. 21.4 ± 14%, for rhamnose and glucose in *S. schenckii* and *S. brasiliensis*, respectively. In both cases, *p* < 0.05; Figure 3). In both species, the wall sugar composition of cells grown in BHI was similar to that found in cells grown in YPD, but YP- and YNB-grown yeast-like cells showed a significant reduction in the rhamnose and mannose content, which was accompanied by an increment in glucose content (Figure 3). For the case of glucosamine, this showed a significant increment in *S. brasiliensis* YP- and YNB-grown cells (Figure 3). Although a similar trend was observed in *S. schenckii* YP- and YNB-grown cells, the glucosamine increment was not statistically significant when compared to cells grown in YPD or BHI (*p* = 0.06, 0.07, 0.13, and 0.18 for YPD vs. YP, YPD vs. YNB, BHI vs. YP, and BHI vs. YNB, respectively; Figure 3). In both species, cells grown in YP and YNB did not show significant differences in any of the four sugars analyzed compared with each other (Figure 3). In the case of the *S. globosa* cell wall, the cells grown in YPD showed significantly lower rhamnose and mannose content when compared to either the *S. schenckii* or *S. brasiliensis* cell walls (Figure 3). The glucosamine levels were similar for the three species, but glucose was significantly higher only when compared to the *S. brasiliensis* cell wall (Figure 3). Although the glucose content in *S. globosa* tended to be higher, this was not significant compared to the *S. schenckii* wall (*p* = 0.07). The *S. globosa* cells grown in any of the four culture media under analysis showed similar rhamnose, glucose, glucosamine, and mannose content (Figure 3).

**Figure 4:**
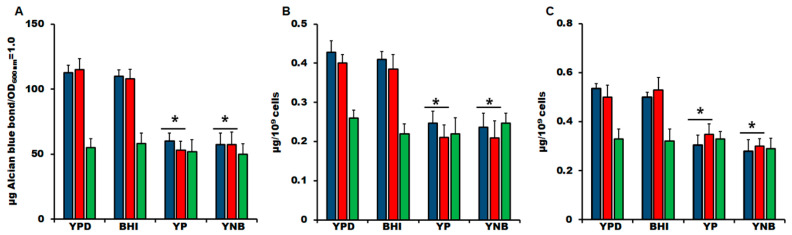
Cell wall binding ability and glycan content of *Sporothrix* spp. yeast-like cells grown in different culture media. Yeast-like cells from *Sporothrix schenckii* (blue bars), *Sporothrix brasiliensis* (red bars), or *Sporothrix globosa* (green bars) were grown in the indicated culture medium and used to calculate the cell ability to bind the cationic dye Alcian blue (**A**) or were treated with either endoglycosidase H (**B**) or β-elimination (**C**) to remove *N*-linked and *O*-linked glycans, respectively. Results are the media ± standard deviation from three independent experiments performed by duplicate. * *p* < 0.05 when compared to the sugar content of YPD- or BHI-grown cells.

The results shown in Figure 3 indicate that YP and YNB stimulated a decrease in mannan and rhamnose content in both *S. schenckii* and *S. brasiliensis*, suggesting a reduction in either the cell wall glycoprotein content or the size of the glycans decorating wall proteins. The first of these hypotheses is unlikely, as the cell wall protein content suffered minimal variations in cells grown in the different culture media (178.8 ± 29.4, 188.8 ± 35.7, and 195 ± 18.7 µg of protein/mg of the cell wall for *S. schenckii*, *S. brasiliensis*, and *S. globosa*, respectively; the values are the means of the cell wall protein content from cells grown in the four culture media under analysis). The ability of fungal cells to bind Alcian blue has been associated with the net negative charge of its surface, which depends on a proper display of both *N*- and *O*-linked glycans on the fungal surface [33,55,57,69,70,71]. Therefore, defects in protein glycosylation often reduce the ability of fungal cells to bind this cationic dye [33,55,57,65,69,70,71]. The YPD- and BHI-grown *S. schenckii* cells bound similar dye levels (Figure 4A), and the mean was similar to that previously reported for cells growing in YPD [33]. However, YP- and YNB grown cells showed about a 50% reduction in the Alcian blue bound, suggesting defects in the protein glycosylation pathways (Figure 4A). A similar trend was observed in *S. brasiliensis* cells, showing high dye content bound in cells growing in either YPD or BHI and a reduction in its binding ability when grown in YP or YNB (Figure 4B). In the case of *S. globosa* cells, they had a lower dye-binding ability compared to *S. schenckii* or *S. brasiliensis* cells (*p* < 0.05 in all cases; Figure 4A), and the Alcian blue bound did not significantly change when cells were grown in any of the four culturing media under study (Figure 4A). Next, *N*-linked and *O*-linked glycans were trimmed by treating cell walls with endoglycosidase H or β-elimination, respectively, and the released sugars quantified as described in the Materials and Methods section. Both *N*-linked and *O*-linked glycan levels showed minimal variation in *S. schenckii* and *S. brasiliensis* cells grown in YPD or BHI, but YP- and YNB-grown cells displayed lower levels of both glycans (Figure 4B,C). In the case of *S. globosa*, the culture medium did not affect the *N*-linked and *O*-linked glycan content, and this was lower when compared to the levels found in *S. schenckii* and *S. brasiliensis* cells (*p* < 0.05, Figure 4B,C). Therefore, YP and YNB culture media affected the wall glycan content in *S. schenckii* and *S. brasiliensis* but not in *S. globosa*.

**Figure 5:**
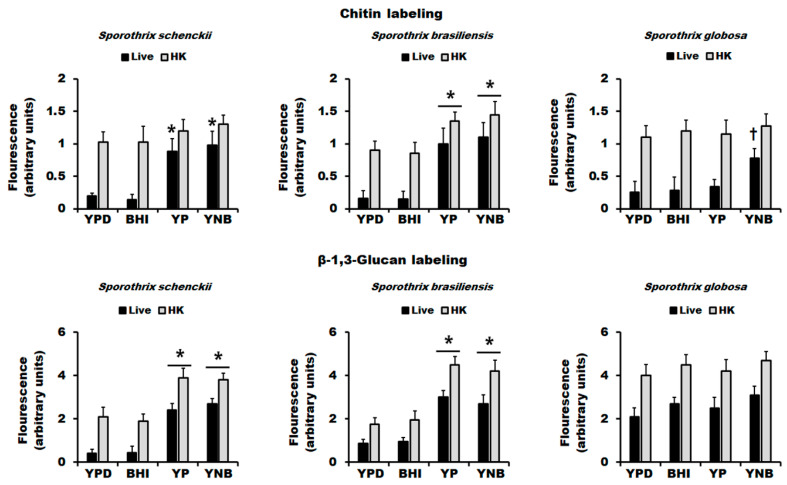
Cell wall chitin and β-1,3-glucan labeling in *Sporothrix schenckii*, *Sporothrix brasiliensis*, and *Sporothrix globosa* growing in different culture media. Yeast-like cells grown in the indicated culturing media were harvested, washed, and labeled with either fluorescein isothiocyanate–wheat germ agglutinin conjugate or IgG Fc-Dectin-1 chimera for chitin and β-1,3-glucan, respectively, and inspected under fluorescence microscopy, and the fluorescence of 300 cells per condition was calculated as described in the Materials and Methods section. Data are represented as mean ± standard deviation of the three independent experiments performed in duplicate. * *p* < 0.05 when compared to the fluorescence associated with YPD- or BHI-grown cells under the same condition. ^†^ *p* < 0.05 when compared to live cells grown in YPD, BHI, or YP medium. HK, heat-killed cells.

It has been previously reported that the main cell wall polysaccharides, named chitin and β-1,3-glucan, are mainly underneath other cell wall components, being barely detectable by fluorescent-conjugated lectins on the surface of both *S. schenckii* and *S. brasiliensis* yeast-like cells [14,33,34]. As reported in other fungal species, the cell killing by heat artifactually exposed both polysaccharides on the cell surface [63,72,73,74], allowing an estimation of the total content of the detected wall component. As previously reported, live *S. schenckii* and *S. brasiliensis* cells grown in YPD showed poor labeling of both polysaccharides, which significantly increased in HK cells (Figure 5). For both fungal species, the cell growth in the BHI medium did not affect the distribution of chitin or β-1,3-glucan in the cell wall (Figure 5). In the case of cell growth in YP or YNB of these two species, there was a significant increment in the labeling of both chitin and β-1,3-glucan in live cells, suggesting a higher presence of both polysaccharides on the wall surface (Figure 5). The fluorescent signals associated with chitin and β-1,3-glucan labeling in YP- or YNB-grown *S. brasiliensis* cells were significantly higher than those found in YPD- or BHI-grown cells (Figure 5), confirming our previous observations generated by HPAEC-PAD, i.e., more chitin and β-1,3-glucan in these cell walls. A similar observation was found in *S. schenckii* cells but only when β-1,3-glucan was labeled (Figure 5). When similar experiments were performed with *S. globosa* cells, we found that most of the chitin was not labeled in live cells grown in YPD, BHI, or YP, suggesting that it is covered by other wall components like in *S. schenckii* and *S. brasiliensis* (Figure 5). Although the chitin labeling of live cells growing in YNB was significantly lower compared with HK cells (*p* < 0.05), this was significantly higher when compared to the fluorescence found in YPD-, BHI, or YP-grown cells (Figure 5). The β-1,3-glucan labeling in live and HK *S. globosa* cells showed no significant variations depending on the culture media, but the labeling in live cells grown in YPD or BHI was significantly higher than the mean fluorescence found in *S. schenckii* or *S. brasiliensis* growing under the same conditions (*p* < 0.05 in all cases), suggesting that the *S. globosa* cells have the β-1,3-glucan more exposed on the cell surface than cells from the other two species when grown in ether YPD or BHI. Taken together, these data indicate that the culture medium to grow *S. schenckii*, *S. brasiliensis,* or *S. globosa* yeast-like cells affected chitin exposure on the cell wall, while β-1,3-glucan distribution was not affected in *S. globosa* cells.

### 3.3. Analysis of Secreted Protease and Lipase/Esterase Activity and Uptake by Hemocytes of Sporothrix schenckii, Sporothrix brasiliensis, and Sporothrix globosa Grown in Different Culture Media

It has been previously reported that β-1,3-glucan exposure on the cell wall surface has a major role in the ability of *S. schenckii* and *S. brasiliensis* yeast-like cells to stimulate TNFα, IL-1α, IL-1β, and IL10 production by human peripheral blood mononuclear cells [14], and the interaction of this wall component with dectin-1 drives the uptake of *Candida* spp. cells and *Aspergillus fumigatus* by human and murine macrophages [64,75,76,77,78]. Therefore, we hypothesized that the increased exposure of β-1,3-glucan on the cell wall surface, as a consequence of the culturing media, could have an impact on the ability of *G. mellonella* hemocytes to uptake *Sporothrix* cells. These immune cells showed a similar ability to uptake *S. schenckii* yeast-like cells grown in either YPD or BHI media, but the fungal uptake was higher when cells were grown in either YP or YNB (Table 2). No significant differences were observed when compared to the uptake of YP- and YNB-grown cells (Table 2). Laminarin has been reported as an antagonist of dectin-1, deactivating the downstream signaling cascades to stimulate cytokine production and phagocytosis [56,64,76,77], and similar observations have been reported in hemocytes from *G. mellonella* [79,80]. Upon hemocytes’ pre-incubation with laminarin, the fungal uptake was significantly reduced and was similar for the cells grown in any of the four culture media analyzed (Table 2). This reduction was more prominent in hemocytes interacting with YP- or YNB-grown cells (Table 2). In the case of *S. brasiliensis*, the uptake of YPD- and BHI-grown cells was similar among them but significantly differed from those observed with cells from *S. schenckii* and *S. globosa* grown under the same culture media (Table 2, *p* < 0.05 in all cases). However, hemocytes showed an increased ability to uptake YP- and YNB-grown cells, and the uptake figures were similar compared with those observed with cells from the two species grown in the same culture media (Table 2). Similar to the observation in the system with *S. schenckii* cells, laminarin significantly reduced the fungal uptake when any of the four culture media were used to grow *S. brasiliensis* cells (Table 2). Finally, when hemocytes were challenged with *S. globosa* cells grown in any of the four culture media, the uptake was similar for the four conditions analyzed and was affected by laminarin in a similar way to that shown for the other two species (Table 2). The uptake of YP- or YNB-grown cells was similar for the three fungal species but was significantly different for cells grown in either YPD or BHI (Table 2). Taken together, these data suggest that the β-1,3-glucan exposure driven by the culture media to grow fungal cells affected the ability of the hemocytes to uptake yeast-like cells.

Next, we assessed whether the culture media had any effect on the secreted hydrolytic enzymes related to *Sporothrix* spp. virulence, particularly protease and lipase/esterase activity [81]. For protease activity, a control reaction with proteinase K was included, whose hydrolytic activity on bovine serum albumin was of 689.2 ± 83.4 µg of aminoacids/h, and this was reduced to 0.02 ± 0.002 µg of aminoacids/h when the enzyme was previously denatured by boiling for 10 min before adding to the reaction. For the case of lipase/esterase activity, a positive control reaction containing lipase from pig pancreas was used to hydrolyze the substrate 4-methylumbelliferyl-oleate, while a negative control reaction included the enzyme previously denatured by heating for 10 min. The positive control reaction showed 237.4 ± 24.8 nmol of methylumbelliferone per minute, while no product was detected in the negative control reaction. When these enzyme activities were measured in cell-free supernatants, protease and lipase/esterase activities were detected in the preparations, but no differences associated with the effect of the culture media or the fungal species were observed (Table 2), suggesting secreted protease and lipase/esterase activity were not affected by the culture media used to grow *Sporothrix* yeast-like cells.

## 4. Discussion

The fungal virulence assessment in animal models is an essential analysis when pathogenicity mechanisms are dissected, virulence factors and determinants are described, and the positive effect of compounds with antifungal properties are evaluated in *in vivo* conditions. Even though the host selection, route of fungal inoculation, and parameters evaluated during the *Sporothrix*–host interaction are presently well standardized, there is a significant variation in the *Sporothrix* cells’ preparations before inoculation into the host, which might impact the outcome of the experimental disease [41,82]. A search within the available scientific literature found that conidia and yeast-like cells are the most frequent cell morphologies used for animal inoculation, but we only included the latter in this study, as conidia were shown to generate erratic killing curves upon inoculation in *G. mellonella* larvae, and the results were not comparable with those generated in mice [32]. BHI is the most popular culture medium to propagate *Sporothrix* spp. [24,25,30,31,40], and although Sabouraud broth [29] and potato-dextrose broth [17] have also been reported as suitable media to induce dimorphism, we did not include them in this study because we could not generate a significant transition from hyphae to yeast-like cells in these media (it was on average less than 15% in both cases), as reported by other groups [17,29]. Instead, we included YPD, another rich medium used to preserve and stimulate *Sporothrix* spp. dimorphism [14,33,34,83]. Moreover, most of the composition and structure analyses of *S. schenckii* and *S. brasiliensis* cell walls have been performed in YPD-grown cells [14,48], and it has been reported that the culture media and growth conditions influence fungal biology, including the cell wall composition [43,45]. Although YP and YNB media are not considered poor media, they contain a relative limitation of carbon and nitrogen sources, respectively, compared with BHI and YPD. Thus, cells growing in YP or YNB are more prone to trigger carbon-limitation and nitrogen-limitation stress signals, respectively, than those growing in richer media. This was evident by the difficulty in undergoing dimorphism for *S. schenckii* and *S. brasiliensis* in YP and YNB media. Interestingly, this was not observed for *S. globosa*, suggesting that this species might have increased metabolic plasticity than *S. schenckii* and *S. brasiliensis*. In line with this observation, the three *Sporothrix* species show a different ability to use sugars as a carbon source, a phenotypical method used for taxonomical purposes [84].

As mentioned, the *G. mellonella* model has been previously standardized to assess the *S. schenckii* and *S. brasiliensis* virulence; thus, there was no prima facie reason to propose that this would be useless to analyze the *S. globosa*-host interaction. Our results clearly showed that the mortality curves associated with the three species portrayed the current virulence ranking for these three species, with *S. globosa* being the one with the slowest ability to kill the animal population and *S. brasiliensis* being the fastest among the three species under analysis. One limitation we have to acknowledge about our study is the inclusion of only one strain per species analyzed, and replicate studies with more isolates, in particular of *S. globosa*, are required to provide strength to our results. Nonetheless, to our knowledge, this is the first report of the implementation of the *G. mellonella* model to assess *S. globosa* virulence. The *S. schenckii* and *S. brasiliensis* virulence significantly changed when grown in either YP or YNB, suggesting that limitation of either carbon or nitrogen may have an impact on the ability to kill larvae. Accordingly, in *C. albicans*, the expression of encoding genes for secreted aspartic proteinases is regulated by both the availability of nitrogen as carbon sources, and fungal adhesion and biofilm formation are affected by the carbon source [85]. Our results also suggest that the metabolic changes induced by the culture media to grow cells do not have permanent or long-lasting duration, as the colony-forming units recovered from infected animals were similar for the three species analyzed and for the four culture media used to prepare the fungal inocula, suggesting the yeast-like cells were capable of adapting to the host milieu in a similar rate and that the outcome of the larvae-fungus interactions depends on early events upon inoculation.

The LDH release as a measure of *G. mellonella* cytotoxicity has been poorly studied when used as a host of fungal or bacterial experimental infections [50,54,86,87]. However, our results showed a correlation between short median survival of animals and higher LDH release, suggesting that the presence of this enzyme activity in the cell-free hemolymph could be a good predictor of the *Sporothrix* virulence.

In the model of experimental candidiasis in *G. mellonella* larvae, it is well documented that highly virulent *Candida* spp. cells stimulate a reduction in circulating hemocytes, at 12 h since the postinoculation time [38,50]. The proposed mechanisms for the reduction in circulating hemocytes are the formation of hemocytes–*Candida* clumps [88] and the protrusion of hyphae from the immune cells. In the latter, upon phagocytosis, the fungal cells pierce the cell membrane and promote hemocyte lysis [39]. Here, however, we observed an inverse relation, with killing curves with short median survival associated with high circulating hemocyte counts, and killing curves with slow mortality rates associated with low hemocyte countings. A similar observation was reported by our group when analyzing the protective effect of recombinant Gp70 on the outcome of *S. schenckii*–*G. mellonella* interaction. One possible explanation for this observation is that *Sporothrix* yeast-like cells, like *C. albicans* yeast cells [89], will not pierce the hemocyte membrane, positively affecting the hemocyte concentration in the hemolymph.

Melanin synthesis is a key process in sclerotization, wound healing, and defense reactions against microorganisms that infect the hemocele [37]. Phenoloxidase catalyzes phenol oxidation to quinones that non-enzymatically polymerize, forming melanin [37]. It has been recently demonstrated that phenoloxidase activity increments after the inoculation of highly virulent *Candida* species, but this activity suffers modest changes when the fungal virulence is attenuated [50]. Recombinant Gp70 harboring the amino acid sequence of *S. schenckii* protein induced an increment in the phenoloxidase activity in a dose-dependent manner [51]. Here, we also observed an association between short median survival of animals with high levels of enzyme activity, suggesting that *G. mellonella* immunological response is common for both *Candida* and *Sporothrix* infections.

The changes in the *S. schenckii* and *S. brasiliensis* cell wall composition are in line with our findings in the *G. mellonella* model, where cells associated with median survival times showed pronounced changes in the wall composition. The lack of significant changes in the *S. globosa* wall composition supports our previous observation about the metabolic plasticity of this organism under the growth conditions analyzed. The YP and YNB had a similar effect on the cell wall composition of both *S. schenckii* and *S. brasiliensis*, inducing the increment of glucan content and the reduction of both *N*-linked and *O*-linked glycan levels. Although both species showed a similar trend in terms of chitin accumulation in both culture media, this was only significant in *S. brasiliensis*. In *Saccharomyces cerevisiae*, it has been reported that cell wall mannan reduction is observed when cells are grown in carbon sources difficult to assimilate, such as sucrose, ethanol, and galactose, and it has been suggested that carbon limitation negatively influences mannan synthesis [90], offering a possible explanation for our observations. Furthermore, the changes in cell wall composition reported here resemble the phenotype of mutants in the protein glycosylation pathways [33,55,58,64,69,70,71,76], and the mechanism behind this observation has been associated with the cell integrity signaling pathway, where Mkc1 is phosphorylated [55,69]. A similar observation has been reported for *C. albicans* growing in lactate, where this kinase is mostly phosphorylated and therefore activated when compared to glucose-grown cells [43]. Although this signaling pathway has not been studied in *Sporothrix* spp., we have recently demonstrated that the putative encoding gene for this protein is found within the *S. schenckii* genome (SPSK_09240) and corresponds to a functional gene that is upregulated in YPD-grown yeast-like cells [91]. Thus, we hypothesize that this signaling pathway is present in *Sporothrix* spp. and is stimulated differentially in *S. globosa* cells. More studies are required to address these points.

The changes in the wall composition also affected polysaccharide exposure at the cell surface, and we demonstrated that at least the β-1,3-glucan exposure diminished the fungal virulence since the *S. schenckii* and *S. brasiliensis* YP- and YNB-grown cells, associated with longer median survival times, were more readily uptaken than BHI- and YPD-grown cells. The involvement of this polysaccharide in the fungal uptake was confirmed by laminarin, an antagonist of GmCP8 and apoLp-III, which are insect proteins that work as pattern recognition receptors for fungal β-1,3-glucan sensing, and that opsonize fungal cells for encapsulation and uptake by hemocytes [79,80]. Therefore, it is tempting to speculate that the growth of *Sporothrix* spp. cells in YP or YNB may have a positive impact on the ability of dectin-1-containing cells to uptake *Sporothrix* yeast-like cells and in the rapid control of the experimental infection in mice. In the case of *S. globosa*, in all the culture media tested, β-1,3-glucan was significantly exposed compared to *S. schenckii* or *S. brasiliensis*, providing a possible explanation for the low virulence reported for this species [19]. This natural exposure of the polysaccharide at the cell surface might be related to the presence of shorter *N*-linked and *O*-linked glycans on the cell wall of this species, generating a thinner outermost wall layer compared with the thicker glycoprotein layer already observed in both *S. schenckii* and *S. brasiliensis* cells [48]. Additionally, it likely suggests that the exposure of this wall component induced by the culturing media could be part of the main early fungal traits that define the outcome of the host–fungus interaction.

The relevance of β-1,3-glucan exposure in the *Sporothrix* spp. virulence place this polysaccharide as an attractive target for the development of immunomodulatory strategies. This cell wall component is located under the peptidorhamnomannan layer [14,48], and the use of drugs capable of exposing larger amounts of β-1,3-glucan by interfering with the synthesis of rhamnose could be helpful to enhance the host immune response against the pathogen.

At first glance, the measurements of secreted protease and lipase/esterase activities did not show variations in the different species and growth conditions, suggesting they may have a marginal impact on the *Sporothrix* virulence. However, since several genes encoding these functions have been predicted within the genome of these organisms [15,81], our strategy cannot discard the particular contribution of any of those gene products in the ability of these organisms to invade *G. mellonella* larvae. Additional studies are required to address this subject.

In conclusion, we reported here that at difference of *S. globosa*, the *S. schenckii* and *S. brasiliensis* virulence is influenced by the culture media where yeast-like cells are prepared, modifying the cell wall composition and exposure of β-1,3-glucan, which positively affected the ability of insect hemocytes to uptake yeast-like cells. These culturing media effects should be taken into consideration when the virulence of *Sporothrix* spp. is analyzed.

## Figures and Tables

**Table 1:** Fungal burden, cytotoxicity, and some immunological parameters of *Galleria mellonella* inoculated with *Sporothrix* spp.

Organism	Colony-Forming Units (×10^5^) ^a^	Cytotoxicity (%) ^b^	Hemocytes (×10^6^)/mL ^c^	Phenoloxidase ^d^
PBS ^e^	0.0 ± 0	13.1 ± 3.7	2.9 ± 0.6	0.7 ± 0.2
*Sporothrix schenckii*				
YPD ^f^	2.2 ± 0.4	77.4 ± 9.8 *	7.8 ± 0.8 *	2.8 ± 0.3 *
BHI ^f^	2.4 ± 0.2	52.7 ± 12.4 *	5.8 ± 0.5 *	2.0 ± 0.3 *
YP ^f^	1.9 ± 0.2	32.4 ± 11.4 ^†^	3.7 ± 0.5 ^†^	1.3 ± 0.2 ^†^
YNB ^f^	2.2 ± 0.2	36.7 ± 9.7 ^†^	3.9 ± 0.3 ^†^	1.3 ± 0.4 ^†^
*Sporothrix brasiliensis*				
YPD	2.3 ± 0.3	89.4 ± 7.6 *	9.2 ± 0.5 *	3.5 ± 0.3 *
BHI	1.8 ± 0.3	60.1 ± 12.7 *	5.1 ± 0.9 *	2.6 ± 0.4 *
YP	2.4 ± 0.4	42.4 ± 12.4 ^†^	3.9 ± 0.3 ^†^	1.6 ± 0.5 ^†^
YNB	2.2 ± 0.4	37.0 ± 9.6 ^†^	4.4 ± 0.6 ^†^	1.5 ± 0.6 ^†^
*Sporothrix globosa*				
YPD	2.2 ± 0.3	33.4 ± 10.4	3.9 ± 0.7	1.5 ± 0.3
BHI	2.0 ± 0.4	34.8 ± 12.4	4.1 ± 0.8	1.2 ± 0.4
YP	2.4 ± 0.5	37.5 ± 8.8	4.2 ± 0.4	1.5 ± 0.6
YNB	1.9 ± 0.5	40.4 ± 11.1	3.6 ± 0.6	1.2 ± 0.2

^a^ Surviving and dead animals were decapitated, and the hemolymph was collected and used to calculate the colony-forming units by serial dilutions of the hemolymph and incubation on YPD plates. ^b^ Measured as the lactate dehydrogenase activity in the cell-free hemolymph retrieved from infected animals. The 100% cytotoxicity corresponds to the enzyme activity quantified from lysed hemocytes. ^c^ The hemolymph from infected animals was collected and used to quantify hemocytes. ^d^ Defined as the Δ_490nm_ per min per μg protein. ^e^ Animal control group inoculated with PBS. ^f^ The culture media where cells were grown. * *p* < 0.05 when compared with the values obtained with the yeast-like cells grown in other culture media from the same species. ^†^ *p* < 0.05 when compared with the values obtained with the yeast-like cells grown in either YPD or BHI from the same species.

**Table 2:** Analysis of secreted protease and lipase/esterase activity and uptake by hemocytes of *Sporothrix schenckii*, *Sporothrix brasiliensis*, and *Sporothrix globosa* grown in different culture media.

Organism	Uptake by Hemocytes (×10^3^ Cells)	Uptake by Hemocytes (×10^3^ cells) + Laminarin ^a^	Protease Activity ^b^	Lipase/Esterase Activy ^c^
PBS ^e^	0.0 ± 0	0.0 ± 0	0.01 ± 0.005	0.0 ± 0.0
*Sporothrix schenckii*				
YPD ^d^	6.5 ± 0.6	3.8 ± 0.8 †	357.4 ± 89.7	38.2 ± 13.5
BHI ^d^	7.4 ± 0.8	3.2 ± 0.6 †	387.1 ± 76.1	42.7± 8.9
YP ^d^	12.9 ± 0.7 *	2.9 ± 1.0 †	366.7 ± 97.4	33.7± 11.4
YNB ^d^	13.4 ± 0.4 *	3.3 ± 0.8 †	438.7 ± 77.5	44.5 ± 10.0
*Sporothrix brasiliensis*				
YPD	3.8 ± 0.2	2.4 ± 0.6 †	402.8 ± 92.1	35.2 ± 6.8
BHI	4.4 ± 0.6	2.5 ± 0.8 †	387.2 ± 47.8	33.6 ± 9.1
YP	10.4 ± 0.7 *	3.1 ± 1.1 †	364.1 ± 67.0	38.4 ± 8.8
YNB	11.2 ± 0.9 *	2.5 ± 1.3 †	442.1 ± 57.9	40.1 ± 10.4
*Sporothrix globosa*				
YPD	11.4 ± 0.8	3.9 ± 0.8 †	357 ± 99.2	40.5 ± 11.4
BHI	12.2 ± 0.7	2.4 ± 1.2 †	380 ± 95.4	35.2 ± 9.7
YP	13.4 ± 0.8	4.1 ± 0.9 †	411 ± 62.7	33.7 ± 10.4
YNB	12.1 ± 0.6	3.7 ± 1.0 †	435 ± 41.8	44.1 ± 8.0

^a^ Hemocytes were preincubated with 200 µg/mL laminarin for 60 min at 37 °C before the incubation with the fungal cells. ^b^ Expressed as µg of aminoacids per 60 min per total secreted protein. ^c^ Expressed as nmol of methylumbelliferone per minute per total secreted protein. ^d^ The culture media where cells were grown.* *p* < 0.05 when compared with the values obtained with the yeast-like cells grown in either YPD or BHI from the same species. ^†^ *p* < 0.05 when compared with the values obtained with no preincubation with laminarin.
